# Humoral Response to SARS-CoV-2-Vaccination with BNT162b2 (Pfizer-BioNTech) in Patients on Hemodialysis

**DOI:** 10.3390/vaccines9040360

**Published:** 2021-04-08

**Authors:** Michael Jahn, Johannes Korth, Oliver Dorsch, Olympia Evdoxia Anastasiou, Burkhard Sorge-Hädicke, Bartosz Tyczynski, Anja Gäckler, Oliver Witzke, Ulf Dittmer, Sebastian Dolff, Benjamin Wilde, Andreas Kribben

**Affiliations:** 1 Department of Nephrology, University Hospital Essen, University Duisburg-Essen, Hufelandstr. 55, 45147 Essen, Germany; johannes.korth@uk-essen.de (J.K.); bartosz.tyzcynski@uk-essen.de (B.T.); anja.gaeckler@uk-essen.de (A.G.); benjamin.wilde@uk-essen.de (B.W.); andreas.kribben@uk-essen.de (A.K.); 2 KfH Kuratorium für Dialyse und Nierentransplantation e.V, KfH-Nierenzentrum Friesener Straße 37a, 96317 Kronach, Germany; oliver.dorsch@kfh.de; 3 Institute for Virology, University Hospital Essen, University Duisburg-Essen, Virchowstr. 179, 45147 Essen, Germany; olympiaevdoxia.anastasiou@uk-essen.de (O.E.A.); ulf.dittmer@uk-essen.de (U.D.); 4 KfH Kuratorium für Dialyse und Nierentransplantation e.V, KfH-Nierenzentrum Alfried-Krupp-Str. 43, 45131 Essen, Germany; burkhard.sorge-haedicke@kfh-dialyse.de; 5 Department of Infectious Diseases, West German Centre of Infectious Diseases, University Hospital Essen, University Duisburg-Essen, Hufelandstr. 55, 45147 Essen, Germany; oliver.witzke@uk-essen.de (O.W.); sebastian.dolff@uk-essen.de (S.D.)

**Keywords:** SARS-CoV-2, mRNA-vaccines, hemodialysis, chronic kidney disease, COVID-19, antibody titers

## Abstract

mRNA-based SARS-CoV-2 vaccines offer a preventive strategy against severe acute respiratory syndrome coronavirus-2 (SARS-CoV-2) infections that is of interest in the care of patients on hemodialysis (HDP). We measured humoral immune responses in 72 HDP after standard vaccination with two doses of the mRNA-based SARS-CoV-2 vaccine BNT162b2 (Pfizer-BioNTech). Antibody responses were evaluated with an anti-SARS-CoV-2 IgG ChemiLuminescent ImmunoAssay (CLIA) two weeks after the second dose. In addition, SARS-CoV-2 IgG was determined in a control of 16 healthy healthcare workers (HCW). The control group of HCW has shown a strong antibody response with a median (MD (Q1; Q3)) antibody titer of 800.0 AU/mL (520.5; 800.0). In comparison to HCW, HDP under 60 years of age responded equally (597.0 AU/mL (410.5; 800.0), *p* = 0.051). However, the antibody responses of the HDP negatively correlated with age (r^2^ = 0.2954 *p* < 0.0001), leading to significantly lower antibody titers in HDP over 60 years (280.0 AU/mL (45.7; 477.0), *p* < 0.0001). To thoroughly understand the immunogenicity of the new mRNA-based vaccines in HDP, longitudinal data on the effectiveness and durability of antibody responses are needed. Modifications of immunization schedules should be considered in HDP with low or without antibody responsiveness after standard vaccination to boost immune reactivity and prolong protective effects in these vulnerable patients.

## 1. Introduction

Patients on hemodialysis (HDP) are at high risk for severe coronavirus disease 2019 (COVID-19)-associated complications [1]. European and Canadian register studies report a 20-30% lethality of HDP with COVID-19, which classifies these patients as a high-risk population in terms of severe acute respiratory syndrome coronavirus-2 (SARS-CoV-2) infections [2,3,4]. Moreover, hygienic concepts in dialysis centers are particularly challenging. In the case of a SARS-CoV-2 infection, HDP cannot entirely fulfill quarantine restrictions, as dialysis treatment cannot be discontinued. Furthermore, HDP often present high dependencies on different other health care institutions, such as nursing homes or high hospitalization rates. Therefore, dialysis centers are at risk to become centers of outbreaks or to facilitate spreading into other critical medical infrastructures. 

Thus, implementations of additional complex hygienic measures are mandatory in dialysis centers but lead also to a significantly increased workload for the dialysis staff. Vaccination is regarded as a cost and a resource-effective preventive measure to reduce individual and institutional risks of both HDP and dialysis centers, which is why a prioritization for vaccination is frequently demanded [5].

However, hyporesponsiveness to vaccination is frequently reported in HDP. Therefore, modifications of standard immunization schedules to boost immune reactivity are applied in these patients and are common practice for, e.g., hepatitis B vaccinations. [6,7,8,9,10,11]. The reasons for the abnormal immunogenicity of different vaccines in HDP are multifactorial. Both the innate and adaptive immune system are disturbed by uremia, leading to insufficient neutrophil and monocyte function, decreased antigen-processing, and reduced cell-mediated and antibody-mediated immune responses [12,13]. Further risk factors like higher age, diabetes, obesity, dialysis vintage, malnutrition, or inflammation were identified to additionally impair protective immunity in HDP after hepatitis B vaccinations [14,15,16,17].

mRNA-based vaccines are a new class of vaccines, and SARS-COV-2 mRNA vaccines were highly efficient in phase III clinical trials [18,19,20,21]. However, the efficacy has not been studied in HDP and it is not known whether mRNA vaccines induce protective immunity in these patients. The aim of this study was to evaluate humoral responses after two vaccinations with BNT162b2—an mRNA-based SARS-CoV2-2 vaccine (BioNTech/Pfizer)—in HDP.

## 2. Materials and Methods

Seventy-two HDP were vaccinated with the mRNA-based SARS-CoV-2 vaccine BNT162b2 (Pfizer-BioNTech) at a vaccination center in Kronach, Germany according to the standard protocol (two doses of 30 µg administered three to four weeks apart) [20]. None of these HDP had immunosuppressive medication or previously reported SARS-CoV-2-infections. Before the first vaccination and fourteen days after the second vaccination, serum samples of 72 HDP were tested for SARS-CoV-2 IgG against the Spike glycoprotein using an approved anti-SARS-CoV-2 IgG CLIA (LIAISON^®^ SARS-CoV-2 TrimericS IgG assay, Diasorin, Saluggia, Italy). According to the manufacturer’s recommendations for the CLIA, an Arbitrary Units per milliliter (AU/mL) ratio of <13.0 was considered to be negative and ≥13.0 to be positive. A conversion of AU/mL to binding antibody units (BAU/mL) that correlate with the WHO standard is possible using the following equation: BAU/mL = 2.6*AU/mL. 800.0 AU/mL (2080 BAU/mL) is the upper limit of quantification without dilution of the CLIA. 

In addition, the antibody response was compared to 16 healthcare workers (HCW) after vaccinations at the University Hospital Essen in January 2021 with the same vaccination, sampling, and testing protocol as the HDP. The HCW received regular testing with teal-time PCR-assays for SARS-CoV-2 RNA from nasal swaps and had no clinical suspicion for SARS-CoV-2 infections throughout the preceding 12 months.

HDP were subdivided in age-related groups: 37–59 years, 60–69 years, 70–79 years, and 80–90 years. Statistical analysis was performed using GraphPad Prism. Kruskall–Wallis Test followed by Dunn’s Multiple Comparison Test was used for the analysis of the non-normally distributed data. Linear regression analysis was applied to determine the correlation between antibody titers and the variables age and duration of hemodialysis-dependency. Results were considered statistically significant when the p-value was below 0.05. 

The study was conducted according to the guidelines of the Declaration of Helsinki and approved by the ethics committee of the Medical Faculty of the University Duisburg-Essen (20-9753-BO).

## 3. Results

**Figure 1:**
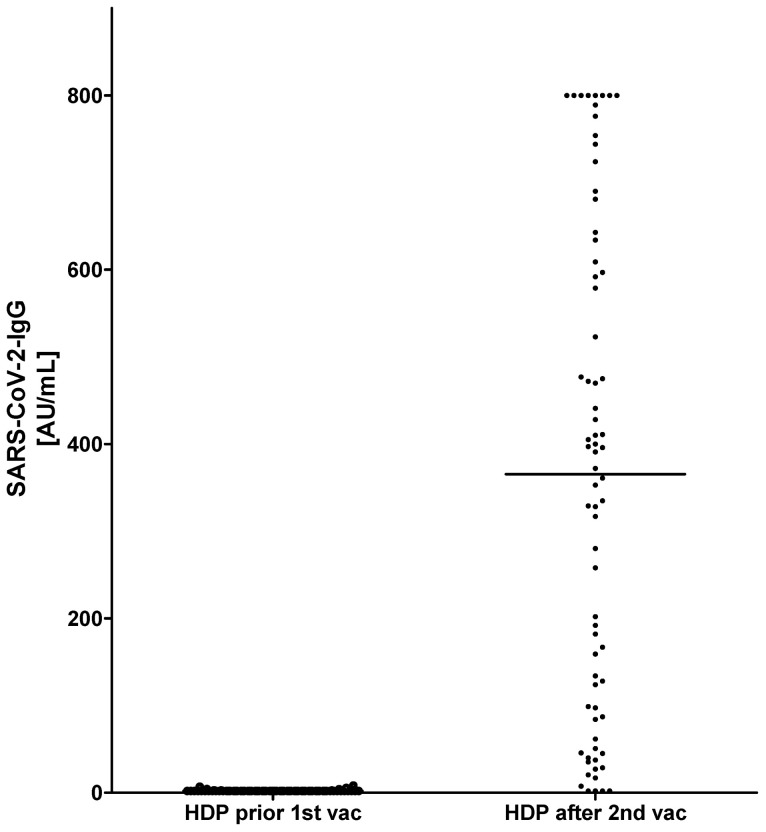
Comparison of antibody titers before and after two vaccinations with mRNA-based severe acute respiratory syndrome coronavirus-2 (SARS-CoV-2) vaccine BNT162b2.

**Figure 2:**
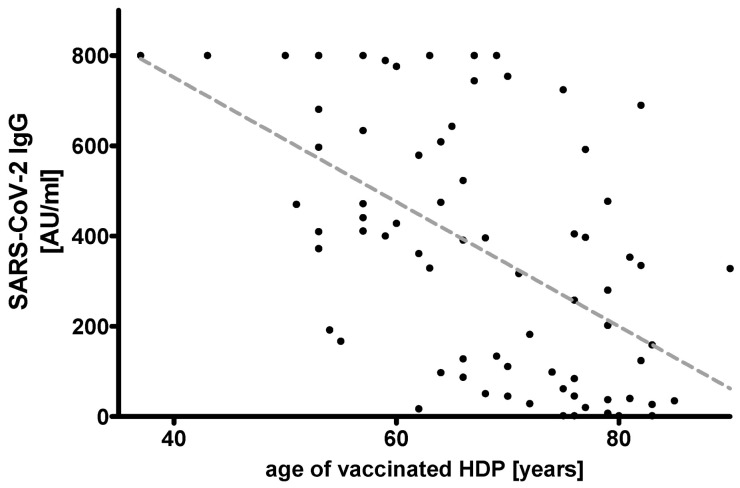
Correlation of age and IgG ChemiLuminescent ImmunoAssay (CLIA) Arbitrary Units per milliliter (AU/mL) ratio in 72 hemodialysis patients (HDP) after two doses of the mRNA-based SARS-CoV-2 vaccine BNT162b2 (r^2^ = 0.2954 *p* < 0.0001).

**Figure 3:**
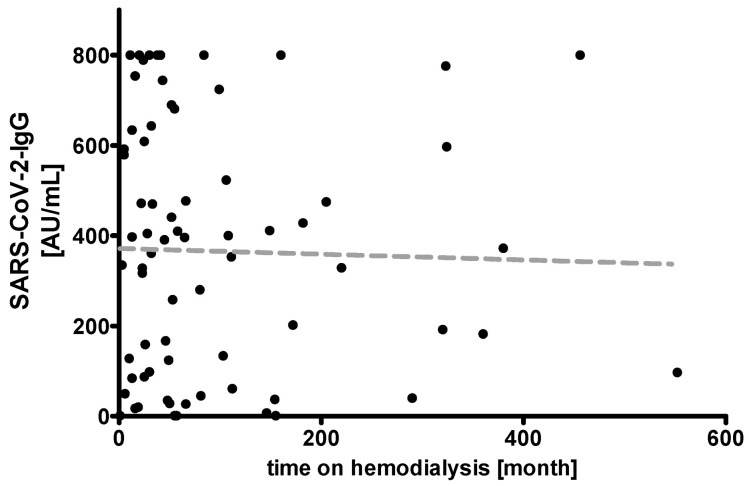
Correlation of duration of hemodialysis-dependency with IgG ChemiLuminescent ImmunoAssay (CLIA) Arbitrary Units per milliliter (AU/mL) ratio in 72 hemodialysis patients (HDP) after two doses of the mRNA-based SARS-CoV-2 vaccine BNT162b2 (r^2^ = 00007, *p* = 0.8261).

All HDP were tested negative for SARS-CoV-2-IgG prior to vaccination (Figure 1). Median (MD (Q1; Q3)) age of all HDP was 68.0 years (60.0; 77.0), median time on hemodialysis was 52.0 month (24.5; 111.7). Two weeks after the second shot, 67 of 72 (93%) HDP were tested positive for SARS-CoV-2 IgG. The median antibody titer in all HDP was 366.5 AU/mL (89.6; 606.0). In HDP, higher age correlated with lower antibody titers (r^2^ = 0.2954, *p* < 0.0001), whereas duration of HD-dependency was not associated with changes in antibody titers (r^2^ = 00007, *p* = 0.8261) (Figure 2 and Figure 3). 

After injection of BNT162b2, only mild localized pain at the injection-site was frequently reported by the HDP, whereas neither severe local reactions like redness or swelling nor systemic reactions like fatigue, headache, chills, fever, muscle, or joint pain were reported for any of the HDP. Thus, overall, the vaccinations were well tolerated. Neither age nor first or second dose of the vaccination played a role in the perception of pain intensity among the HDP. No SARS-CoV-2 infections were reported 13 weeks after the first vaccination in any of the HDP presented here.

Median age of HCW was 45.5 years (41.2–54.7), and median antibody titer was 800.0 AU/mL (520.5; 800.0). Antibody titers were detected in all tested HCW (16 of 16, 100%).

**Figure 4:**
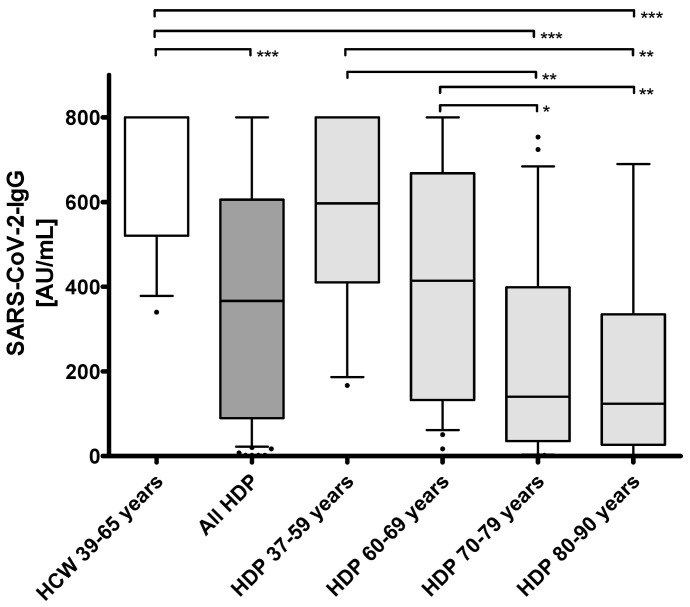
IgG ELISA Arbitrary Units per milliliter (AU/mL) ratio in 72 hemodialysis patients (HDP) and 16 healthcare workers (HCW) after two doses of the mRNA-based SARS-CoV-2 vaccine BNT162b2. Statistical analysis: Kruskal–Wallis Test followed by Dunn’s Multiple Comparison Test, * = *p* < 0.05, ** = *p* < 0.01, *** = *p* < 0.001.

Age and antibody titers were not significantly different between the HCW and youngest group of HDP (37–59 years) with median ages of 45.5 years (41.2–54.7) vs. 54.0 years (53.0; 57.0) (*p* = 0.0716) and median antibody titers of 800.0 AU/mL (520.5; 800.0) vs. 597.0 (410.5; 800.0) AU/mL (*p* = 0.0510) (Table 1, Figure 2). However, seroresponses were consecutively lower with increasing age, which was particularly evident between HDP of 60–69 years and 70–79 years with median antibody titers of 414.0 AU/mL (132.5; 668.3) and 140.0 AU/mL (35.3; 399.0), respectively (*p* < 0.05) (Table 1, Figure 4). 

## 4. Discussion

This is the first study to analyze the humoral response on the mRNA-based vaccine BNT162b2 in patients on hemodialysis (HDP). None of the HDP included to this study showed SARS-CoV-2 specific antibodies before the first vaccination, even though seven cases of COVID-19 were reported from the treating dialysis center. This highlights the effectiveness of hygiene measures. The vast majority of HDP developed specific humoral immune responses upon vaccination (67 of 72; 93%). Similarly high rates of immune responses were reported in healthy participants three weeks after a first dose [22] and one [23] or two [18] weeks after a second dose of 30 µg BNT162b2 by using different SARS-CoV-2 neutralization assays. However, the healthy subjects in these studies were much younger with median or mean ages of 46 years [22], 37.5 years [23], and 36.5 years [18] compared to the median age of 68 years in our chronically ill HDP. This is an important characteristic considering the negative correlation of antibody responses with higher age (Figure 2 and Figure 4) in our cohort. Applying the identical vaccination protocol, Walsh et al. described reduced but still efficient humoral responses of neutralizing antibodies in 12 healthy adults aged 65-74 years as compared to the younger adults in their study [18]. 

The immunogenicity of the mRNA-based vaccine BNT162b2 in chronically ill patients was also tested by Geisen et al [23]. They measured neutralizing and total anti-SARS-CoV-2 IgG in patients (median age 48 years) with chronic rheumatic diseases receiving therapies like TNF blockade or conventional disease-modifying antirheumatic drugs. Similar to our study results, all patients presented antibody titers above the ELISA cut-off but at significantly lower levels compared to a control group of HCW [23].

Limitation of our study is the lack of control groups of older ages. Subsequently, we cannot explicitly attribute the hyporesponsiveness of the elderly HDP to an effect of either senescence or hemodialysis treatment or the combination of both. 

However, this limitation might be subordinate for practical considerations. In contrast to younger adults, most of the currently used vaccines have to be applied in modified schedules in elderly people to improve immunogenicity. This implies higher antigen doses, administration via alternative routes, or the use of adjuvants [11,13,24].

Therefore, the current “one fits all” strategy of COVID-19 vaccination (e.g., two doses of 30 µg BNT162b2 within 3 to 6 weeks) warrants special attention in elderly HDP. Of note, the mean age of HDP in Germany is 68 years [25]. The excellent efficacy of mRNA-based COVID-19 vaccines in healthy subjects, showing greater than 90% efficacy for preventing symptomatic infection after two doses administered 3 to 4 weeks apart (18–21) should not be expected to the same extend in vaccinated HDP, as some of them will stay at high risk for complicated COVID-19 infections due to insufficient seroprotection combined with high vulnerability. Likewise, the duration of vaccine-induced immunity, its efficacy, and its persistence need to be studied. Based on these findings, personalized immunization schedules of elderly HDP need to be considered to boost and sustain immune reactivity. Till we have clarified these concerns, hygienic measures remain an indispensable cornerstone in prevention of SARS-CoV-2 infections in dialysis centers.

## 5. Conclusions

This is the first study describing humoral responses of patients on hemodialysis after vaccination with the mRNA vaccine BNT162b2. Two weeks after the second vaccination with BNT162b2, the vast majority developed specific humoral responses (93%). However, antibody titer negatively correlated with age. Bearing in mind the generally low immunogenicity of vaccines in patients on hemodialysis, personalized immunization schedules in especially elderly patients on hemodialysis should be considered depending on further longitudinal data.

## Figures and Tables

**Table 1:** Characteristics of hemodialysis patients (HDP) and healthy controls of health care worker (HCW).

					Subgroups of HDP							
	HCW 39–65 years		All HDP 37–90 years		HDP 37–59 years		HDP 60–69 years		HDP 70–79 years		HDP 80–90 years	
	MD (Q1; Q3), (range: min–max); or *n* (%)		MD (Q1; Q3), (range: min–max); or *n* (%)		MD (Q1; Q3), (range: min–max); or *n* (%)		MD (Q1; Q3), (range: min–max); or *n* (%)		MD (Q1; Q3), (range: min–max); or *n* (%)		MD (Q1; Q3), (range: min–max); or *n* (%)	
**Subjects**	16		72		17		22		22		11	
**Sex**	♀	9 (56.2%)	♀	31 (43.1%)	♀	8 (47.0%)	♀	10 (45.5%)	♀	8 (36.4%)	♀	4 (36.4%)
	♂	7 (43.8%)	♂	41 (56.9%)	♂	9 (53.0%)	♂	12 (54.5%)	♂	14 (63.6%)	♂	7 (63.6%)
**Age (years)**	45.5 (41.2; 54.7), (range: 39.0–65.0)		68.0 (60.0; 77.0), (range: 37.0–90.0) ***		54.0 (53.0; −57.0), (range: 37.0–59.0) *ns*		64.5 (62.0; 67.0), (range: 60.0–69.0) *		76.0 (73.5; 77.5), (range: 70.0–79.0) ***		82.0 (81.0; 83.0), (range: 80.0–90.0) ***	
**Time on hemodialysis (months)**	-		52.0 (24.5; 111.7), (range: 1.0–552.0)		52.0 (27.0; 240.0), (range: 11.0–456.0)		44.0 (23.7; 126.5), (range: 5.0–552.0)		56.0 (22.0; 102.2), (range: 5.0–360.0)		49.0 (23.0; 111.0), (range: 1.0–290.0)	
**Time between 1st and 2nd vac (days)**	22.0 (22.0; 22.0), (range: 22.0–22.0)		21.0 (21.0; 21.0), (range: 21.0–21.0)		21.0 (21.0; 21.0), (range: 21.0–21.0)		21.0 (21.0; 21.0), (range: 21.0–21.0)		21.0 (21.0; 21.0), (range: 21.0–21.0)		21.0 (21.0; 21.0), (range: 21.0–21.0)	
**Time between 2nd vac and sampling (days)**	13.0 (13.0; 13.0), (range: 13.0–19.0)		17.0 (15.0; 18.0), (range: 15.0–26.0)		17.0 (15.0; 18.0), (range: 15.0–18.0)		17.5 (15.0; 18.0), (range: 15.0–26.0)		17.0 (15.0; 18.0), (range: 15.0–20.0)		15.0 (15.0; 18.0), (range: 15.0–18.0)	
**Ab SARS-CoV-2 CLIA (AU/mL)**	800.0 (520.5; 800.0), (range: 340.0–800.0)		366.5 (89.6; 606.0), (range: 1.8–800.0) ***		597.0 (410.5; 800.0), (range: 167.0–800.0) *ns*		414.0 (132.5; 668.3), (range: 17.0–800.0) *ns*		140.0 (35.3; 399.0), (range: 1.8–754.0) ***		124.0 (27.0; 335.0), (range: 1.8–690.0) ***	

HCW = healthcare workers; HDP = patients on hemodialysis; MD = Median; Q1 = first quartile, Q3 = third quartile; n = count; vac = vaccination; Ab = antibody, CLIA = ChemiLuminescent ImmunoAssay; AU = Arbitrary Units; mL = milliliter. Statistical analysis: Kruskall–Wallis Test followed by Dunn’s Multiple Comparison Test, *ns* = not significant compared to HCW, * = *p* < 0.05 compared to HCW, ** = *p* < 0.01 compared to HCW, *** = *p* < 0.001 compared to HCW.

## Data Availability

The data that support the findings of this study are available from the corresponding author upon reasonable request.
